# Supplementary material for: Probabilistic computing using Cu0.1Te0.9/HfO2/Pt diffusive memristors
Source: Nat Commun. 2022 Sep 30;13:5762. doi: 10.1038/s41467-022-33455-x (PMC9525628; doi:10.1038/s41467-022-33455-x)
Supplement: Supplementary file 1 — Supplementary Information [file 41467_2022_33455_MOESM1_ESM.pdf]

SUPPLEMENTARY INFORMATION

Probabilistic computing using $Cu_{0.1}Te_{0.9}/HfO_2/Pt$ diffusive memristors

Kyung Seok Woo[1], Jaehyun Kim[1], Janguk Han[1], Woohyun Kim[1], Yoon Ho Jang[1] and Cheol Seong Hwang[1*]

[1]Department of Materials Science and Engineering and Inter-University Semiconductor Research Center, Seoul National University, Gwanak-ro 1, Daehag-dong, Gwanak-gu, Seoul, 08826, Republic of Korea.

[#] These authors contributed equally: Kyung Seok Woo, Jaehyun Kim

[*]Corresponding author (e-mail: cheolsh@snu.ac.kr)

Supplementary Figures

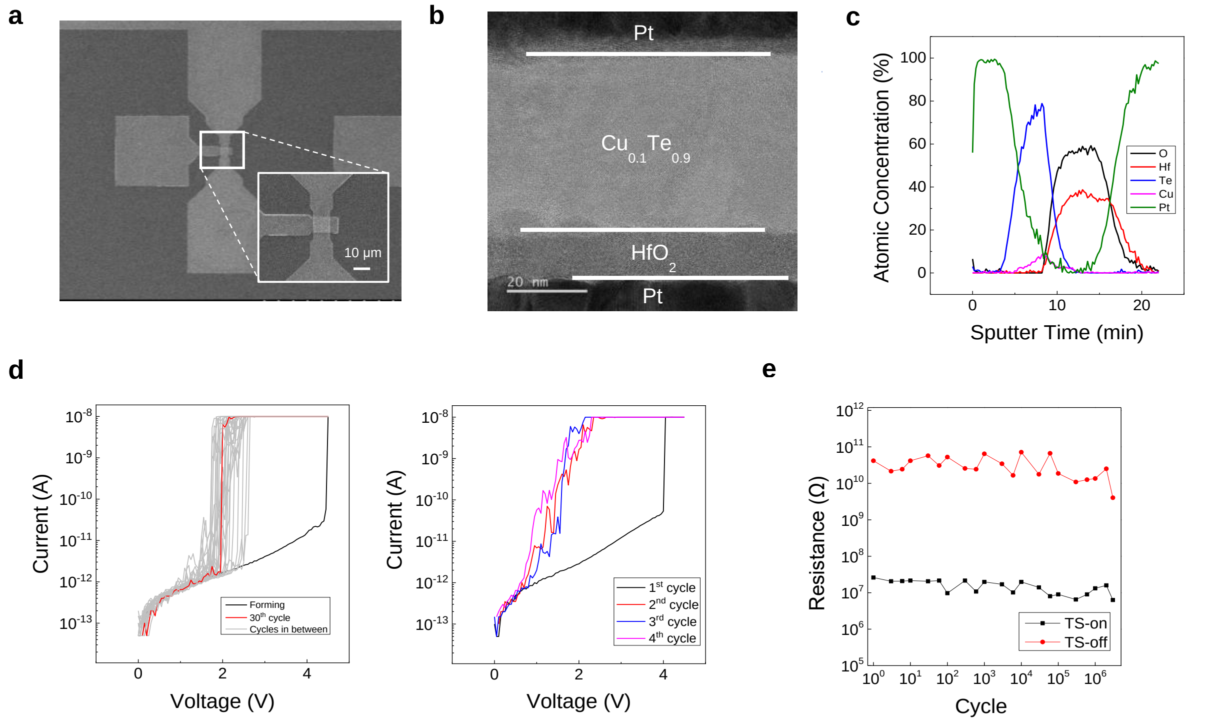

Supplementary Figure S1: a) SEM image of the cross-point structure. b) Cross sectional high-resolution TEM image. c) AES depth profile of the CTHP device. d) I-V curves of TS (left panel, x = 0.1) and RS (right panel, x = 0.2) behaviors of the Cu_xTe_{1-x}-based memristor.[1] e) Cycling endurance of the CTHP device.

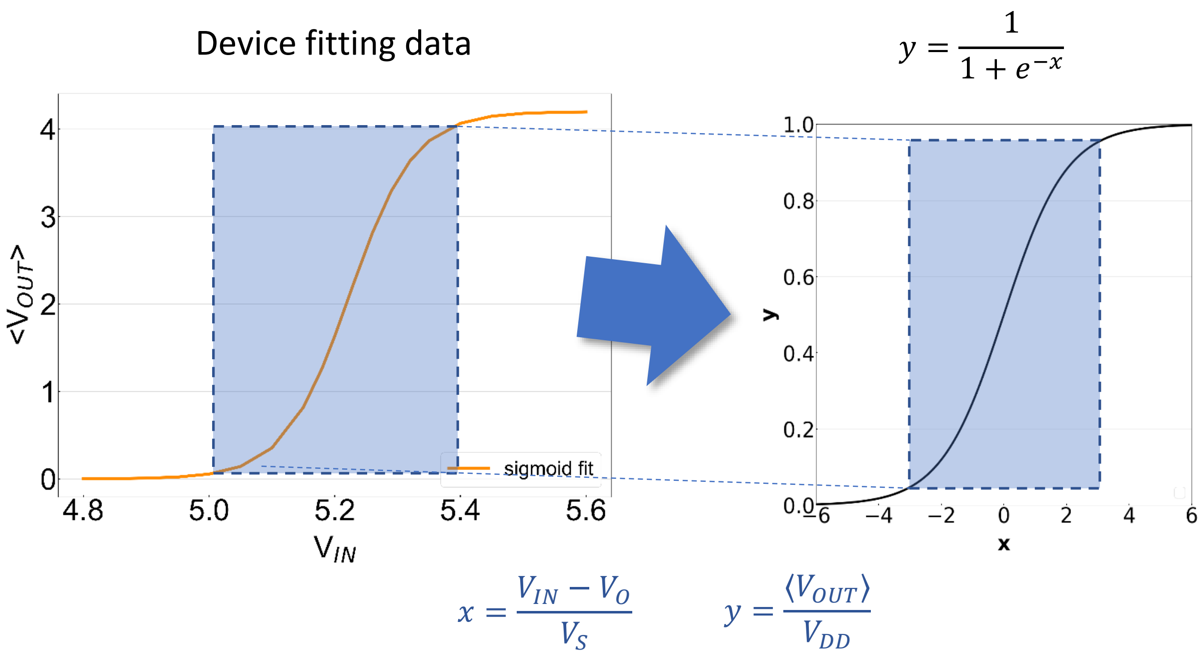

Supplementary Figure S2: Normalization of the device fitting data to sigmoid function.

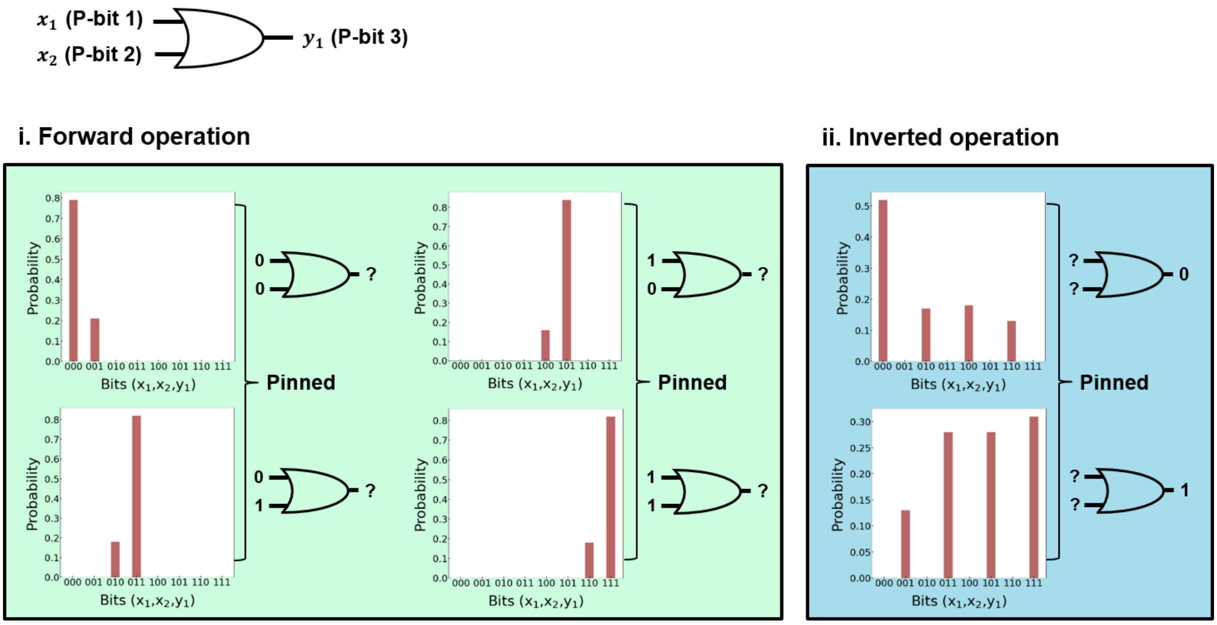

Supplementary Figure S3: x_1 OR x_2. Forward and reverse operations are shown in panels i and ii, respectively.

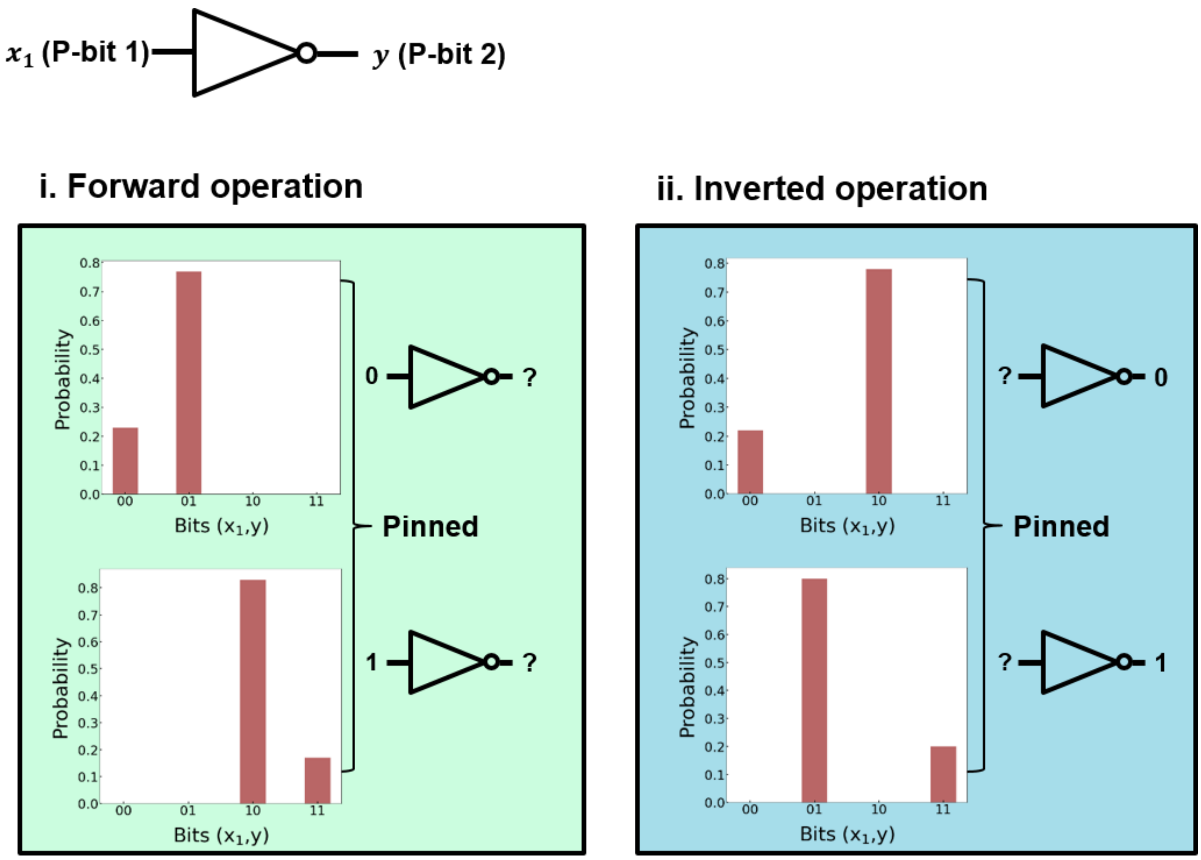

Supplementary Figure S4: NOT x_1. Forward and reverse operations are shown in panels i and ii, respectively.

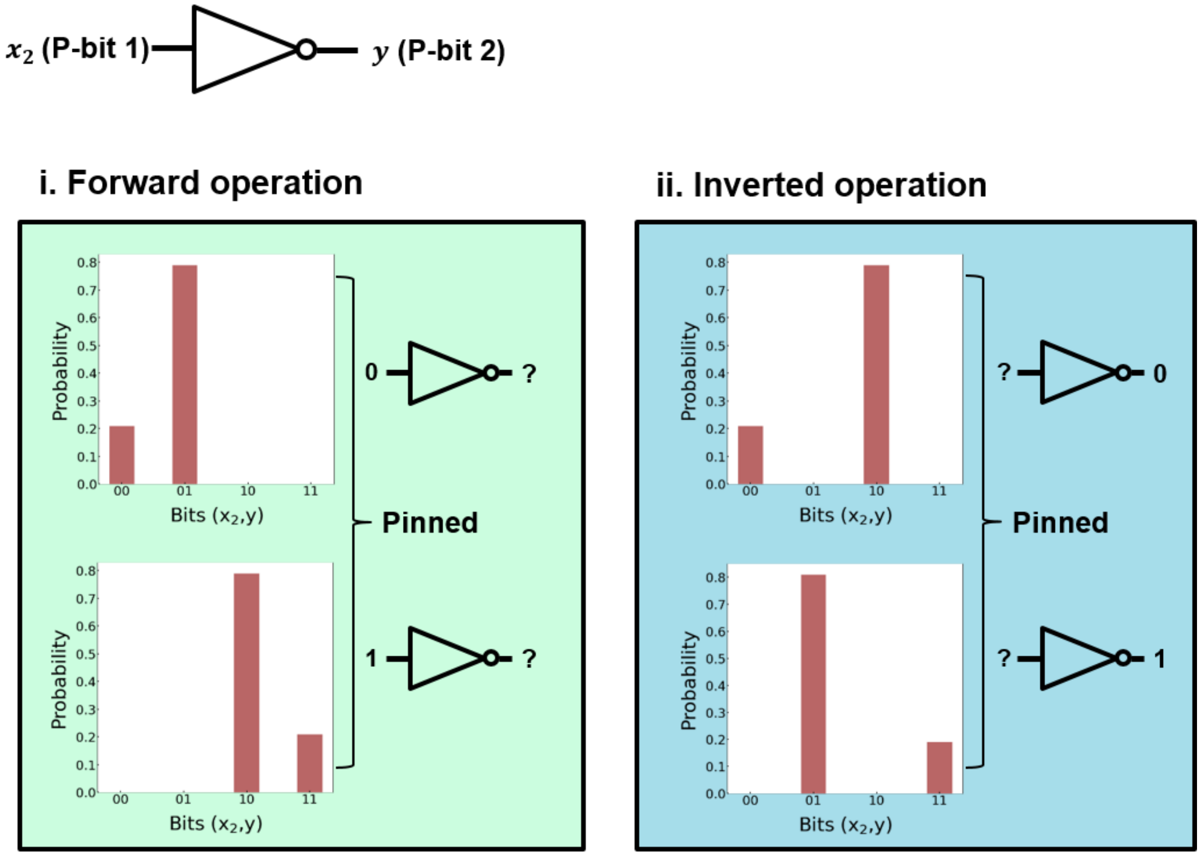

Supplementary Figure S5: NOT x_2. Forward and reverse operations are shown in panels i and ii, respectively.

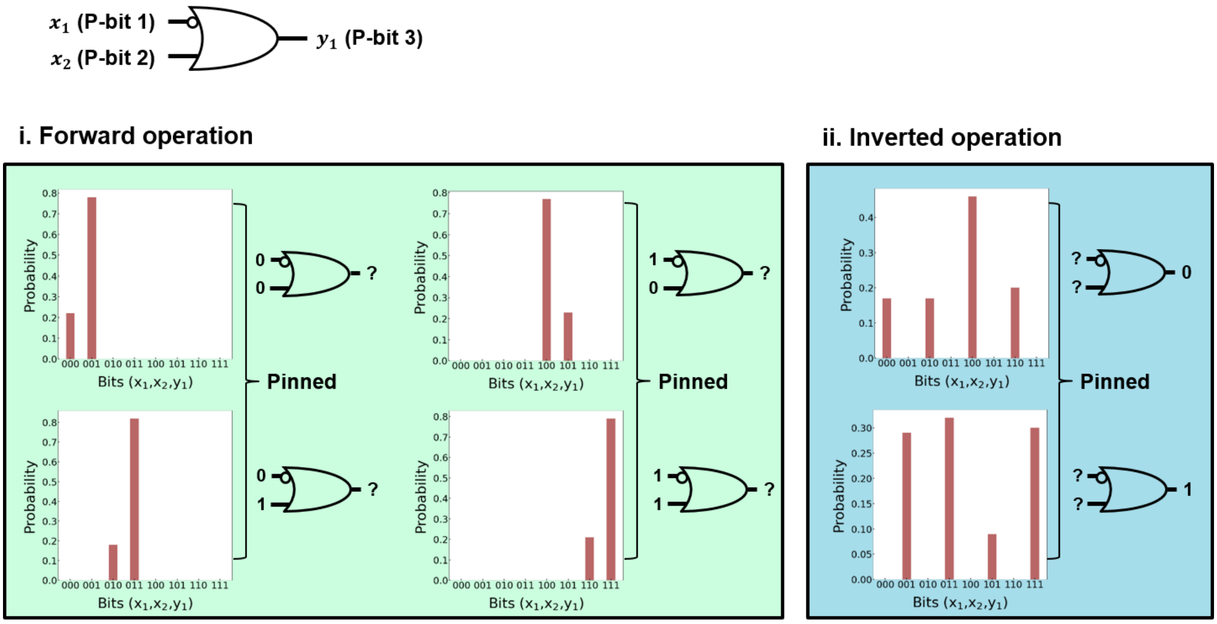

Supplementary Figure S6: x_1 IMP x_2. Forward and reverse operations are shown in panels i and ii, respectively.

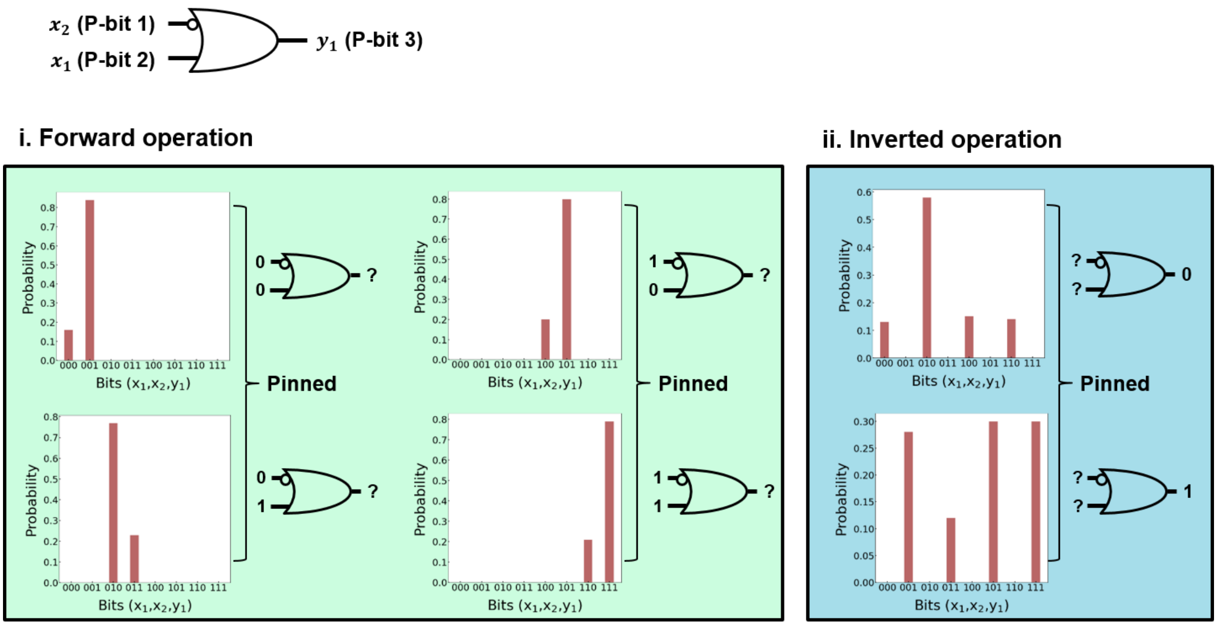

Supplementary Figure S7: x_2 IMP x_1. Forward and reverse operations are shown in panels i and ii, respectively.

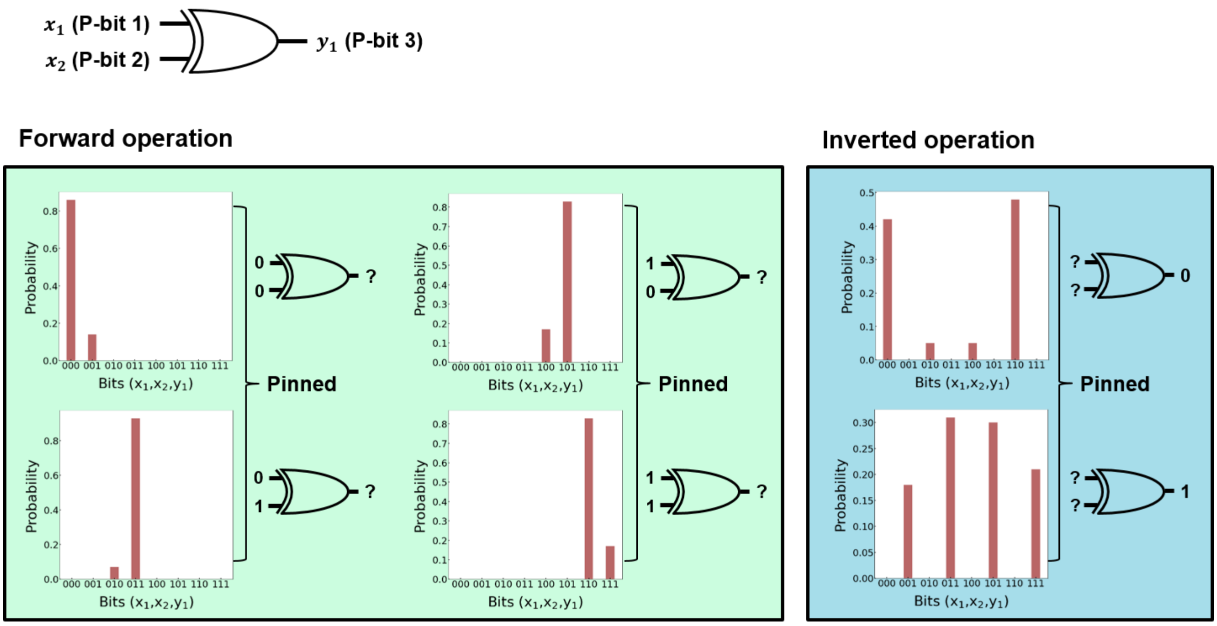

Supplementary Figure S8: x_1 XOR x_2. Forward and reverse operations are shown in panels i and ii, respectively.

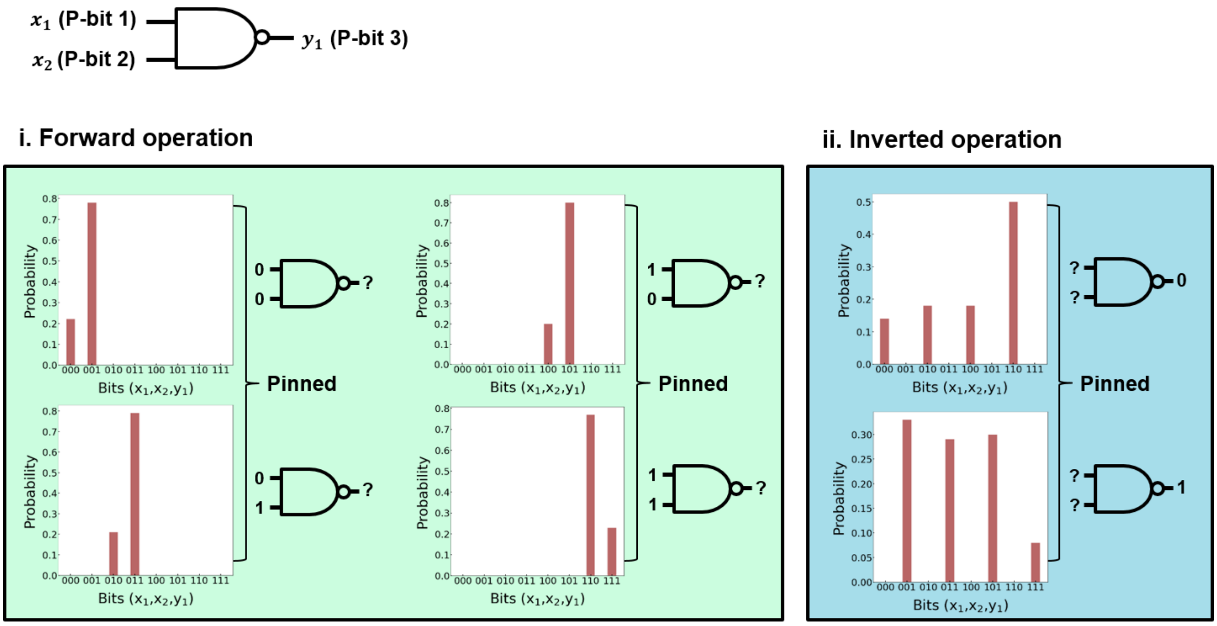

Supplementary Figure S9: x_1 NAND x_2. Forward and reverse operations are shown in panels i and ii, respectively.

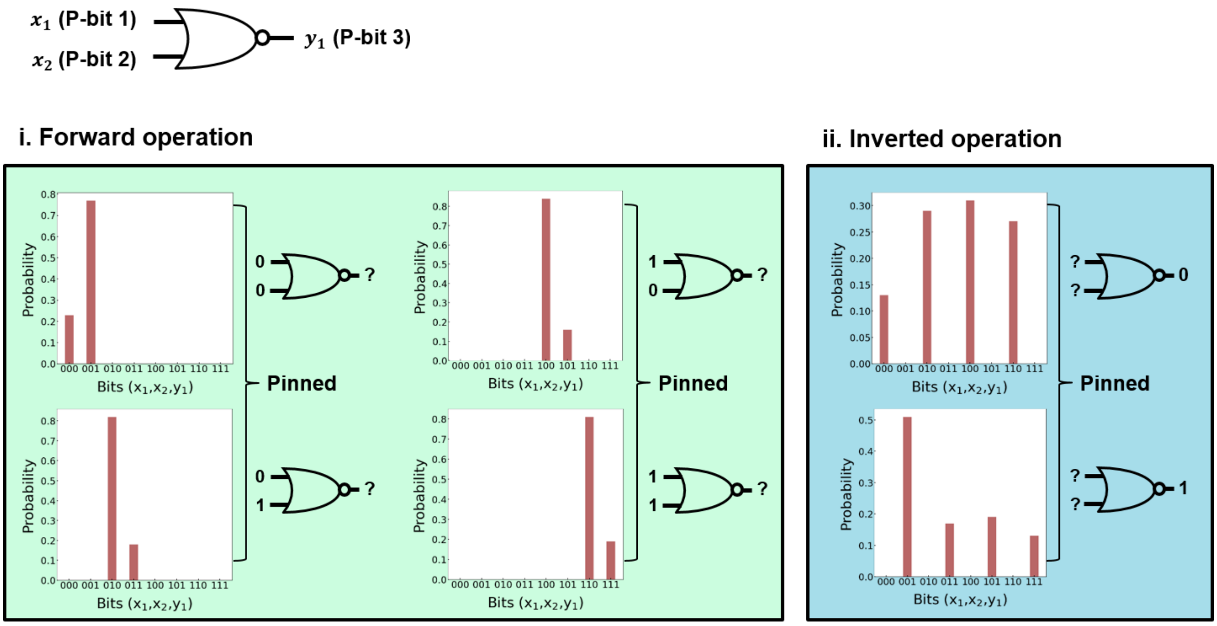

Supplementary Figure S10: x_1 NOR x_2. Forward and reverse operations are shown in panels i and ii, respectively.

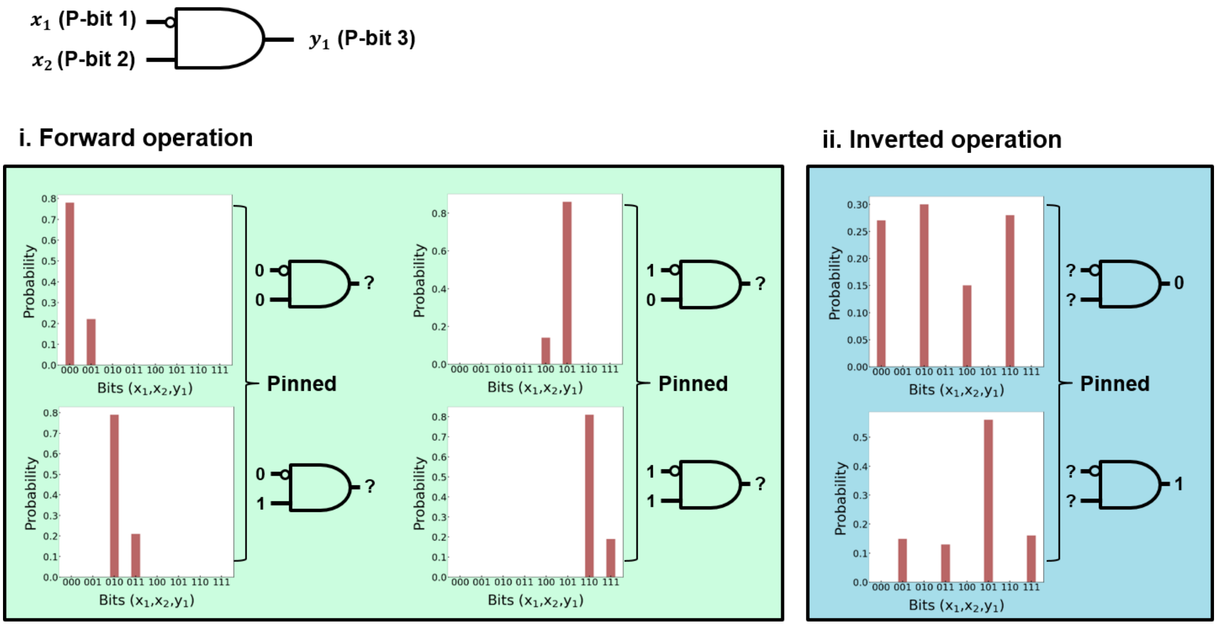

Supplementary Figure S11: x_1 NIMP x_2. Forward and reverse operations are shown in panels i and ii, respectively.

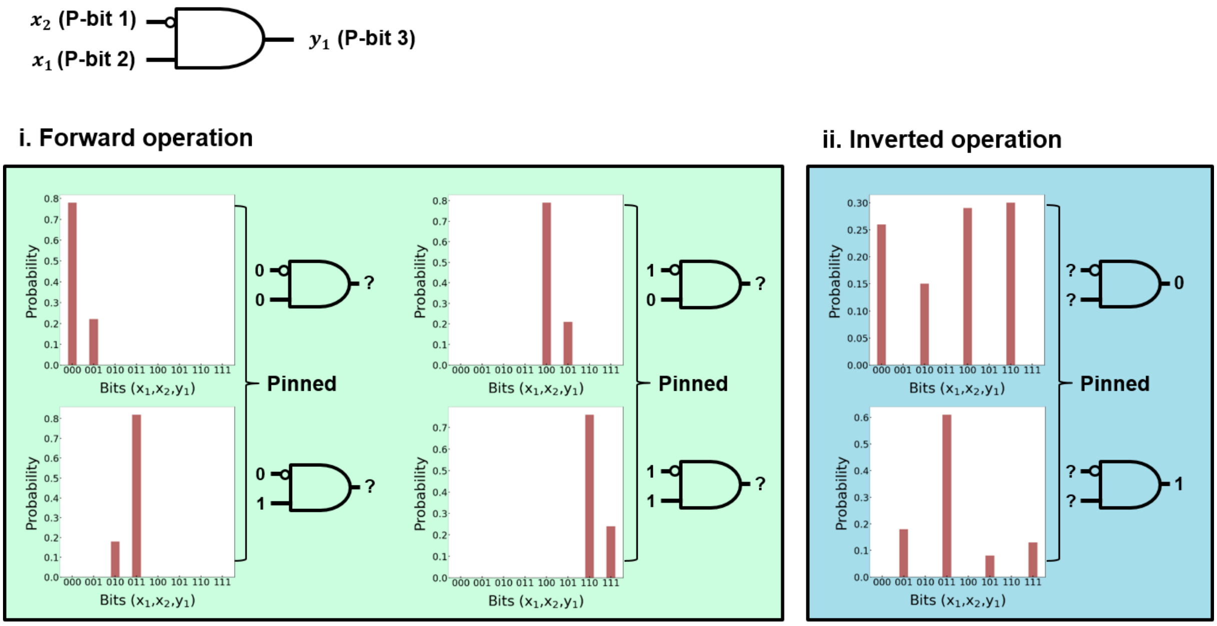

Supplementary Figure S12: x_2 NIMP x_1. Forward and reverse operations are shown in panels i and ii, respectively.

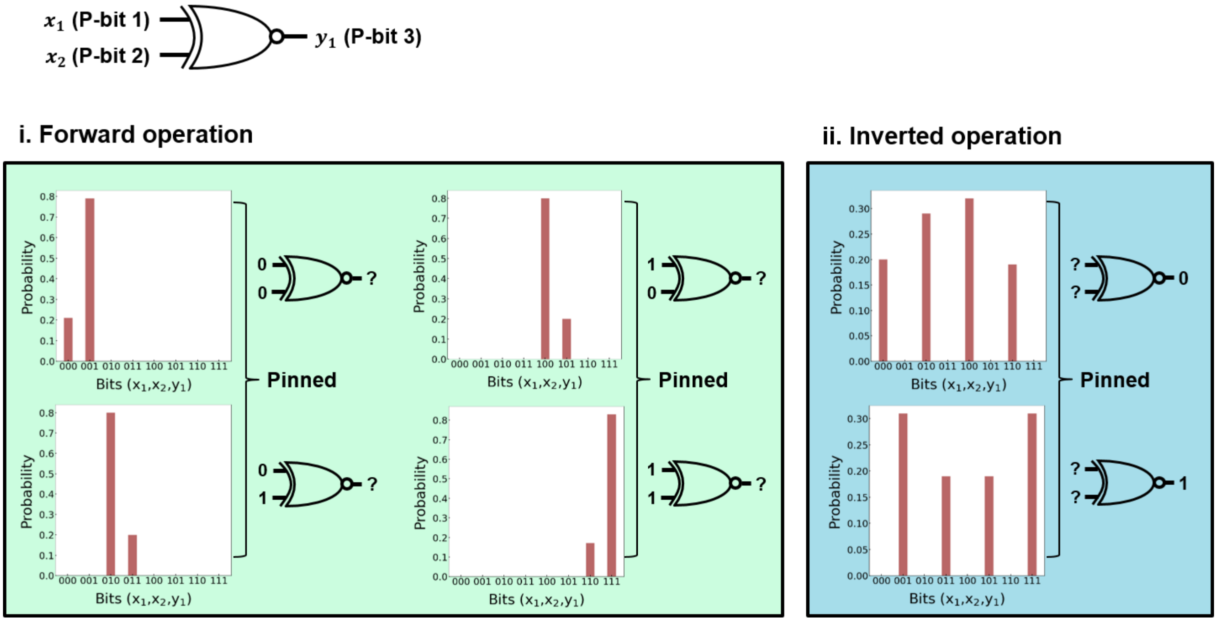

Supplementary Figure S13: x_1 XNOR x_2. Forward and reverse operations are shown in panels i and ii, respectively.

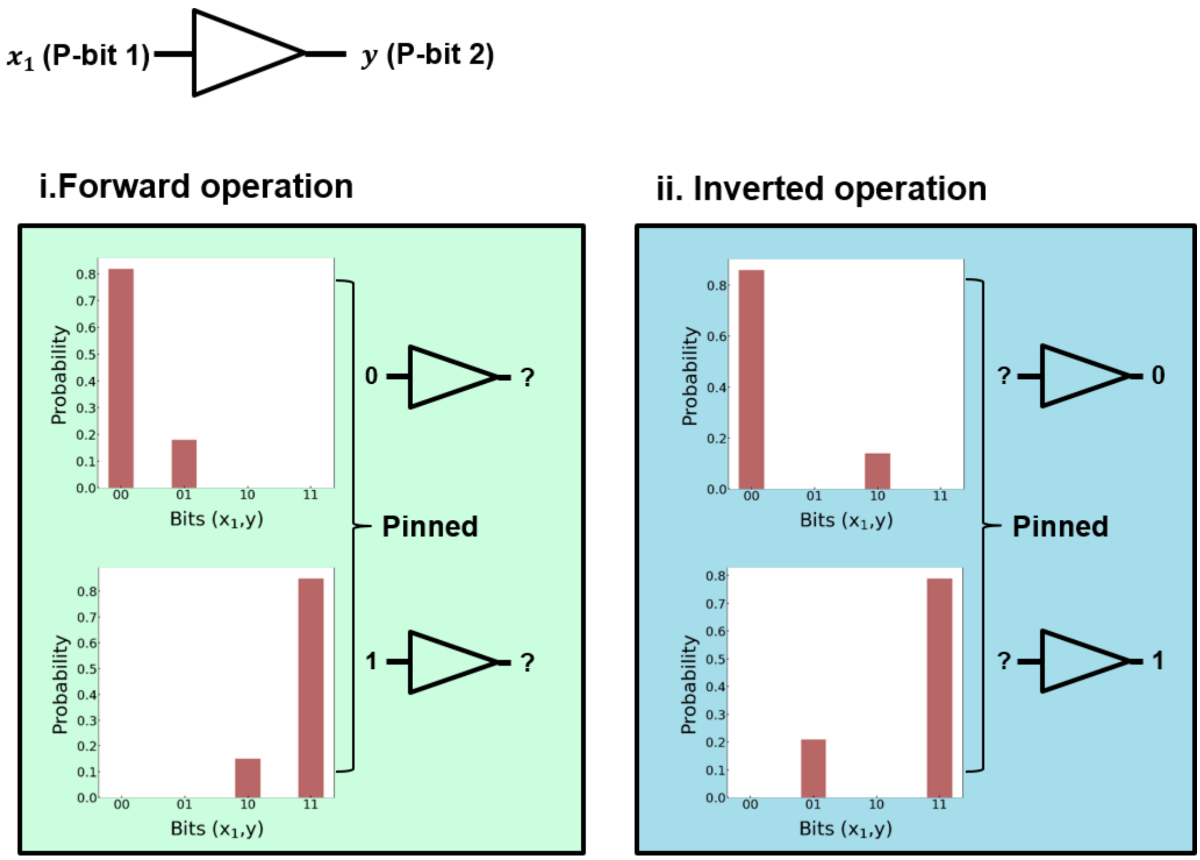

Supplementary Figure S14: x_1. Forward and reverse operations are shown in panels i and ii, respectively.

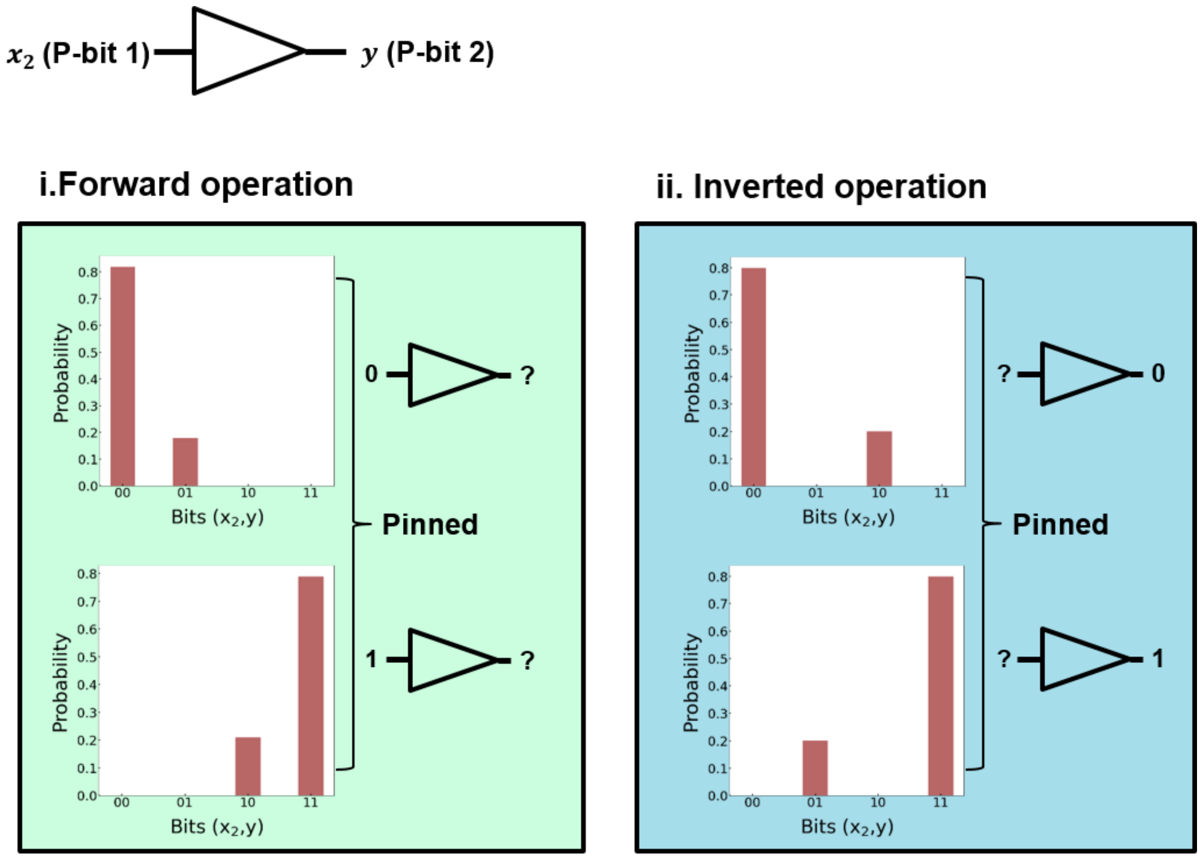

Supplementary Figure S15: x_2. Forward and reverse operations are shown in panels i and ii, respectively.

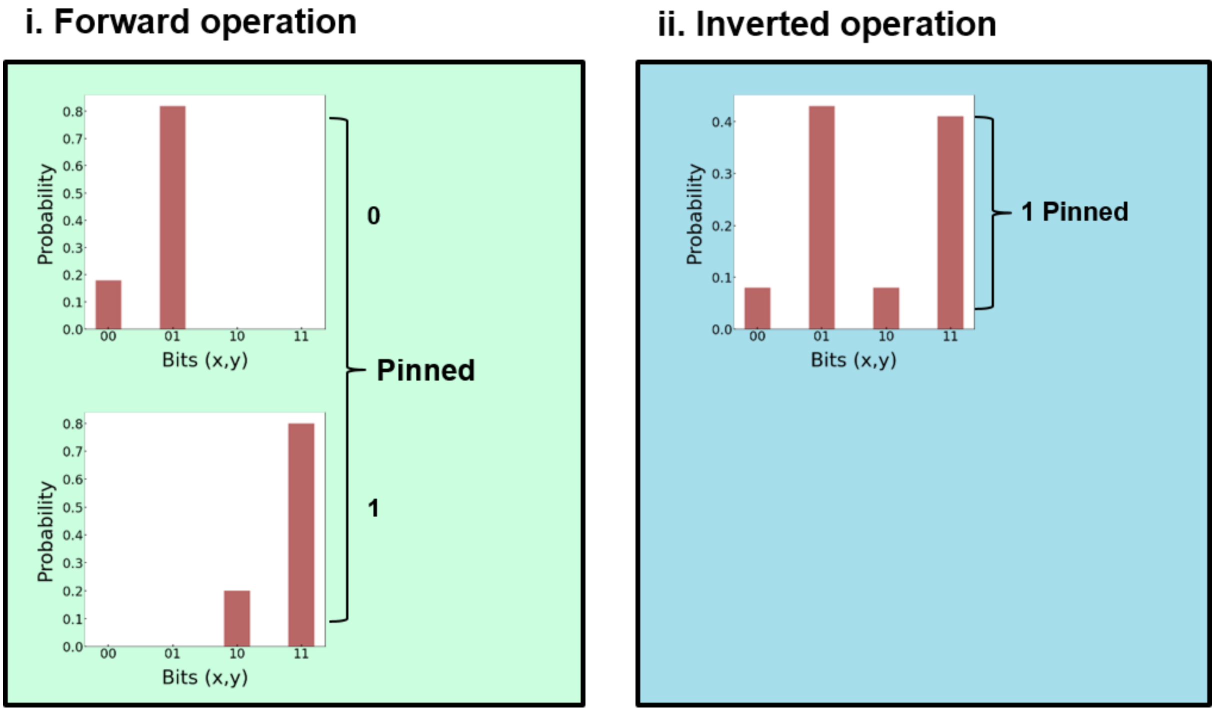

Supplementary Figure S16: TRUE. Forward and reverse operations are shown in panels i and ii, respectively.

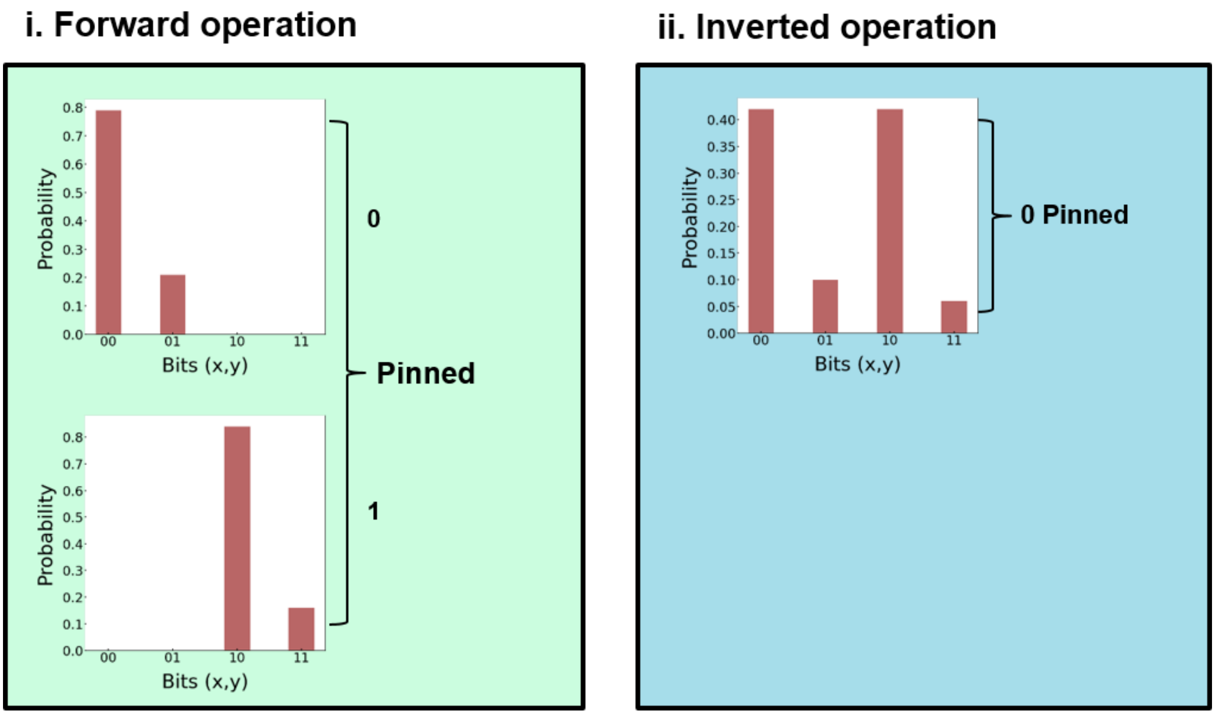

Supplementary Figure S17: FALSE. Forward and reverse operations are shown in panels i and ii, respectively.

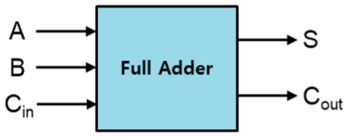

A	B	C_{in}	S	C_{out}
0	0	0	0	0
0	0	1	1	0
0	1	0	1	0
0	1	1	0	1
1	0	0	1	0
1	0	1	0	1
1	1	0	0	1
1	1	1	1	1

$$O + S = A + B + I$$

$$E = I_0(O + S - A - B - I)^2$$

$$E(C_P, \dots, C_1, S_Q, \dots, S_0, A_R, \dots, A_0, B_T, \dots B_0, I_U, \dots I_0) = I_0\left[\left(\sum_{p=1}^{P} 2^p C_p\right) + \left(\sum_{q=0}^{Q} 2^q S_q\right) - \left(\sum_{r=0}^{R} 2^r A_r\right) - \left(\sum_{t=0}^{T} 2^t B_t\right) - \left(\sum_{u=0}^{U} 2^u I_u\right)\right]^2$$

Supplementary Figure S18: A design for a full adder using a five-p-bit network.

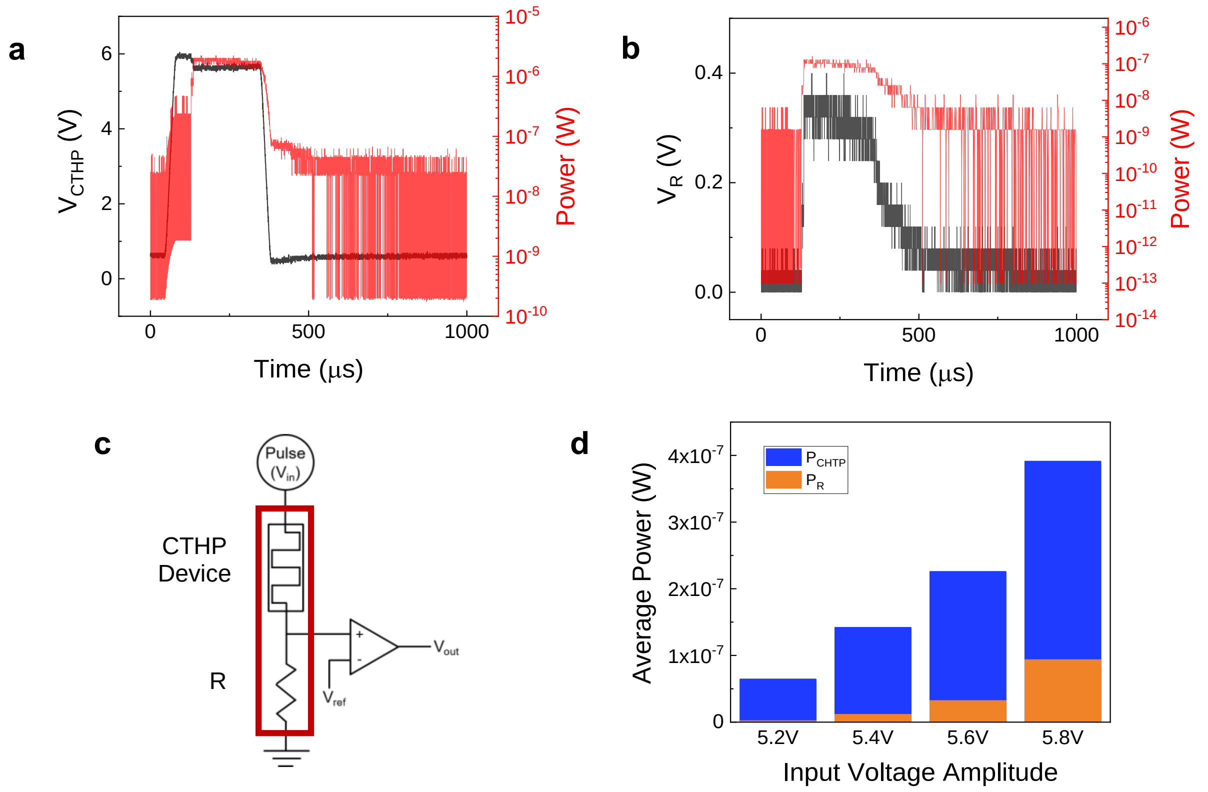

Supplementary Figure S19: Power consumption calculation results of the CTHP p-bit circuit. a) Power consumption of the CTHP device. b) Power consumption of the series resistor. c) The CTHP p-bit circuit design used in the analysis. d) Power consumption at different input voltage pulse amplitudes, averaged over 40 cycles.

Supplementary Tables

Supplementary Table 1: Definitions of cost functions and the resulting input functions for all 16 Boolean logic operations. (I_0 = 1)

**Logic functions**	**Definitions of cost functions**	**Input functions**
AND	$y_1 = x_1x_2$<br>$E(x_1,\ x_2, y_1) = (x_1x_2 - y_1)^2$<br>$= x_1^2x_2^2 - 2x_1x_2y_1 + y_1^2 = x_1x_2 - 2x_1x_2y_1 + y_1$	$I_{x1} = -x_2 + 2x_2y_1$<br>$I_{x2} = -x_1 + 2x_1y_1$<br>$I_{y1} = 2x_1x_2 - 1$
OR	$y_1 = x_1 + x_2 - x_1x_2$<br>$E(x_1,\ x_2, y_1) = (x_1 + x_2 - x_1x_2 - y_1)^2$<br>$= x_1 - 2x_1y_1 + x_2 - 2x_2y_1 - x_1x_2 + 2x_1x_2y_1 + y_1$	$I_{x1} = -1 + 2y_1 + x_2 - 2x_2y_1$<br>$I_{x2} = -1 + 2y_1 + x_1 - 2x_1y_1$<br>$I_{y1} = -2x_1 + 2x_2 - 2x_1x_2 - 1$
NOT	$y = 1 - x$<br>$E(x,\ y) = (x + y - 1)^2$<br>$= 2xy - x - y + 1$	$I_x = -2y + 1$<br>$I_y = -2x + 1$
IMP	$y_1 = (1 - x_1) + x_2 - (1 - x_1)x_2$<br>$= 1 - x_1 + x_1x_2$<br>$E(x_1,\ x_2, y_1) = (1 - x_1 + x_1x_2 - y_1)^2$<br>$= 1 - x_1 + x_1x_2 - y_1 + 2x_1y_1 - 2x_1x_2y_1$	$I_{x1} = -1 + 2x_2 + 2y_1 - 4x_2y_1$<br>$I_{x2} = 2x_1 - 4x_1y_1 - 1 + 2y_1$<br>$I_{y1} = 2x_1 - 4x_1x_2 + 2x_2 + 1$
XOR	$y_1 = (x_1 - x_2)^2$<br>$= x_1 - 2x_1x_2 + x_2$<br>$E(x_1,\ x_2, y_1) = (x_1 - 2x_1x_2 + x_2 - y_1)^2$<br>$= x_1 - 2x_1x_2 - 2x_1y_1 + 4x_1x_2y_1 + x_2 - 2x_2y_1 + y$	$I_{x1} = -1 + 2x_2 + 2y_1 - 4x_2y_1$<br>$I_{x2} = 2x_1 - 4x_1y_1 - 1 + 2y_1$<br>$I_{y1} = 2x_1 - 4x_1x_2 + 2x_2 + 1$
NAND	$y_1 = 1 - x_1\text{AND}x_2$<br>$= 1 - x_1x_2$<br>$E(x_1,\ x_2, y_1) = (1 - x_1x_2 - y_1)^2$<br>$= 1 - x_1x_2 + 2x_1x_2y_1 - y_1$	$I_{x1} = x_2 - 2x_2y_1$<br>$I_{x2} = x_1 - 2x_1y_1$<br>$I_{y1} = -2x_1x_2 + 1$
NOR	$y_1 = 1 - x_1\text{OR}x_2$<br>$= 1 - x_1 - x_2 + x_1x_2$<br>$E(x_1,\ x_2, y_1) = (1 - x_1 - x_2 + x_1x_2 - y_1)^2$<br>$= 1 - x_1 + 2x_1y_1 - x_2 + 2x_2y_1 + x_1x_2 - 2x_1x_2y_1 - y_1$	$I_{x1} = 1 - 2y_1 - x_2 + 2x_2y_1$<br>$I_{x2} = 1 - 2y_1 - x_1 + 2x_1y_1$<br>$I_{y1} = -2x_1 - 2x_2 + 2x_1x_2 + 1$
NIMP	$y_1 = 1 - x_1\text{IMP}x_2$<br>$= x_1 - x_1x_2$<br>$E(x_1,\ x_2, y_1) = (x_1 - x_1x_2 - y_1)^2$<br>$= x_1 - x_1x_2 + y_1 - 2x_1y_1 + 2x_1x_2y_1$	$I_{x1} = -1 + x_2 + 2y_1 - 2x_2y_1$<br>$I_{x2} = x_1 - 2x_1y_1$<br>$I_{y1} = -1 + 2x_1 - 2x_1x_2$
XNOR	$y_1 = 1 - x_1\text{XOR}x_2$<br>$= 1 - x_1 + 2x_1x_2 - x_2$	$I_{x1} = 1 - 2x_2 - 2y_1 + 4x_2y_1$<br>$I_{x2} = -2x_1 + 4x_1y_1 + 1 - 2y_1$

	$E(x_1, x_2, y_1) = (1 - x_1 + 2x_1x_2 - x_2 - y_1)^2$<br>$= 1 - x_1 + 2x_1x_2 + 2x_1y_1 - 4x_1x_2y_1 - x_2 + 2x_2y_1 - y$	$I_{y1} = -2x_1 + 4x_1x_2 - 2x_2 + 1$
x<br>(TRANSFER)	$y = \mathrm{x}$<br>$E(\mathrm{x}, y) = (\mathrm{x} - y)^2$<br>$= \mathrm{x}^2 - 2\mathrm{x}y + y^2$<br>$= \mathrm{x} - 2\mathrm{x}y + y$	$I_x = -1 + 2x$<br>$I_y = -1 + 2x$
TRUE	$y = 1$<br>$E(y) = (1 - y)^2$<br>$= 1 - 2y + y^2$<br>$= 1 - y$	$I_x = 0$<br>$I_{y1} = 1$
FALSE	$y = 0$<br>$E(y) = (-y)^2$<br>$= y^2$<br>$= y$	$I_x = 0$<br>$I_y = -1$

Supplementary Note 1. Ising model

According to the Ising model, the total energy of the spin system is defined by Ising Hamiltonian,

$$E = -\sum_{(i,j)} J_{ij} x_i x_j - \sum_i h x_i \tag{S1}$$

, where x_i, x_j are spins of each site having values of either -1 or 1, J_{ij} is an interaction strength between two spins, and h is the external magnetic field.[2] The first term stands for the internal interaction energy between the spins, and the second term is the energy of the individual spins interacting with the external field. The probability of the system to have the energy value E is calculated by the Boltzmann distribution, $P_i = \exp(-\beta E_i)/Z$, where E_i is the energy of the system, β is $(kT)^{-1}$, and Z is a partition function, $\sum_i \exp(-\beta E_i)$.[3] Accordingly, the spin configuration with the lowest energy occurs with the highest probability. The underlying idea of the Ising model is further extended to a Hopfield network in the machine learning field.

Supplementary References

1. Woo, K. S. *et al.* A High-Speed True Random Number Generator Based on a CuxTe1−x Diffusive Memristor. *Adv. Intell. Syst.* **3**, (2021).
2. Neal, R. M. Connectionist learning of belief networks. *Artif. Intell.* **56**, 71–113 (1992).
3. Camsari, K. Y., Faria, R., Sutton, B. M. & Datta, S. Stochastic p-bits for invertible logic. *Phys. Rev. X* **7**, 31014 (2017).
